# Can We Improve the Prediction of Early Onset Mania and Hypomania in the Community?

**DOI:** 10.1111/bdi.70140

**Published:** 2026-06-25

**Authors:** Jan Scott, Jacob J. Crouse, Sarah E. Medland, Brittany L. Mitchell, Nathan A. Gillespie, Nicholas G. Martin, Ian B. Hickie

**Affiliations:** ^1^ Brain and Mind Centre The University of Sydney Sydney Australia; ^2^ Institute of Neuroscience Newcastle University Newcastle UK; ^3^ Brain and Mental Health Program QIMR Berghofer Institute of Medical Research Brisbane Australia; ^4^ Institute of Molecular Bioscience The University of Queensland Brisbane Australia; ^5^ School of Psychology The University of Queensland Brisbane Queensland Australia; ^6^ School of Psychology and Counselling Queensland University of Technology Brisbane Queensland Australia; ^7^ Virginia Institute for Psychiatric and Behavioral Genetics Virginia Commonwealth University Richmond Virginia USA

**Keywords:** bipolar disorder, boosting, family history, onset, polygenic scores, prediction, youth

## Abstract

**Background:**

There is broad agreement that the onset of bipolar disorders (BD) can be predicted by using combined estimates of familial, genetic and clinical risk. However, there is a lack of consensus about the operationalisation of different risk attributes (e.g., symptoms vs. sub‐threshold syndromes; disorder‐specific polygenic risk scores [PRS] vs. multiple‐disorder PRS dimensions) and their utility for predicting bipolar type 1 (BD‐I) and 2 (BD‐II). Likewise, analyses often fail to consider the optimal model for predicting outcomes where true cases will be in the minority.

**Methodology:**

Proof of concept study employing an ensemble machine learning approach (Boosting) to develop models for classifying BD cases vs. non‐cases using different combinations of risk attributes extracted from a database from a prospective longitudinal follow‐up of twin and non‐twin siblings in the peak age range for onset of major mental disorders.

**Results:**

Of 1473 participants (mean age 26.3; female = 866), 104 developed BD‐I (*n* = 30) or BD‐II (*n* = 74). The best performing Boosting classification had an overall area under the receiver operating curve (AUROC) of 85.1% (95% Confidence Intervals: 80%, 88%); correctly identifying 86.7% BD cases. Variables with greatest relative influence were, in rank order: depressive symptoms, psychotic symptoms, a BD‐Schizophrenia PRS dimension, hypomanic symptoms, and family history of BD. The model accurately classified 89% of manic cases but only 68% of hypomanic cases.

**Conclusions:**

Improving the accurate prediction of BD onset would benefit from greater consensus regarding the operationalisation of known risk attributes and selection of analytic models that consider sample imbalances.

## 1 Introduction

The worldwide prevalence of bipolar disorders (BD) is about 1%–3% and the peak age range for first onset is typically 15–25 years, with some evidence of earlier age at onset in those with a family history of BD [1, 2]. Bipolar‐II disorder (BD‐II) is more common in females, but the initial full‐threshold mood episode in both BD‐I and II is usually depression. Many people who develop BD experience periods with manic or depressive symptoms that are subthreshold for a mood episode diagnosis. Identification of those at high risk of BD remains challenging as the early expressions of psychopathology do not *specifically* predict a BD trajectory [3, 4]. Experts concur that the optimal prediction of BD onset will rely on combined estimates of genetic, familial and clinical risk, but there is less agreement on the most useful estimates to include, or the optimal approach for determining model accuracy in scenarios with low transition rates such as community or primary care samples.

Bipolar disorders have a large hereditary component. A recent meta‐analysis estimated that offspring of parents with BD have a 5% lifetime risk of developing BD and a 55% lifetime risk of developing other mental disorders [5]. Moreover, a *multi*‐*generational* family history of BD or other severe mental disorders is associated with increased likelihood of being diagnosed with BD (and other mental disorders) at an earlier age [6]. Clinically, the utility of family history (FH) has often been undermined by the difficulty with accurate ascertainment [7]. This has led to increased interest in objective (molecular) measures of genetic risk. However, currently, it is unclear whether genetic markers are best used instead of, or as an adjunct to, clinical reports of familial loading [8]. Genome‐wide association studies (GWAS) have demonstrated associations between polygenic liability for BD (i.e., polygenic risk scores; PRS) and later development of BD [9]. Also, a case–control study of PRS in youth with BD (compared with offspring at high‐risk of BD and healthy controls), showed that PRS‐BD was significantly elevated in BD cases, with the suggestion that PRS‐BD demonstrated diagnostic specificity [10]. However, Hafeman et al. [11] argue that PRS‐BD alone has limited utility as a risk predictor at the individual level, and studies show that familial and genetic risk for BD is not uniquely associated with development of BD. For example, PRS‐BD can be associated with the onset of psychosis; and the PRS for schizophrenia (PRS‐SCHIZ), depression (PRS‐MDD) and anxiety or neuroticism (PRS‐NEU) are associated with a small but measurable increase in the likelihood of BD onset in individuals at increased clinical risk for a mood or psychotic disorder, or individuals with a prior early onset depression [5, 12, 13, 14, 15, 16]. Interestingly, studies have demonstrated that PRS estimates may be higher in individuals with more complex or severe mental disorders compared with those who seek help for their condition [15, 17] This is relevant to BD as the performance of prediction models may differ according to help‐seeking status (i.e., clinical versus non‐clinical samples) or in those who experience mania versus hypomania [18].

Genetic variants predisposing individuals to clinically related conditions, such as mood and psychotic disorders, are shared [19]. Given these findings, several researchers have used combined estimates of PRS for more than one severe mental disorder (e.g., dimensional scores for BD‐SCHIZ) as an alternative to disorder‐specific PRS estimates in prediction models [13]. This approach has particularly found support from researchers studying children and adolescents, as during these developmental phases, illness trajectories are more dynamic and may evolve and diverge over time [20, 21]. In sum, it is timely to explore whether there are any additional gains from using different FH constructs (e.g., FH‐BD versus multigenerational‐FH) or using dimensional approaches to PRS (compared with separate estimates of PRS‐BD, PRS‐SCHIZ, etc.).

Research consistently identifies that a range of mood symptoms and subthreshold syndromes may precede the onset of the index full‐threshold hypomanic, manic or depressive episode [3, 4, 22, 23]. Likewise, *psychotic* symptoms are frequently reported by those who later develop BD [24, 25]. Examination of such mood and non‐mood clinical antecedents and sub‐threshold syndromes preceding the onset of full‐threshold episodes of BD has the advantage of mirroring the ultra‐high‐risk categories used in early intervention is psychosis, but the potentially wider range of high‐risk syndromes in BD is a disadvantage [26]. The selection of assessment instruments and use of continuous or categorical measures of early expressions of psychopathology is not only influenced by the psychometric and clinimetric characteristics of the tool, but also by researcher preference (e.g., for specific tools), and the resourcing and nature of the study (e.g., prospective cohort studies, offspring versus healthy controls or other case–control studies, community versus clinical populations, etc. [27]).

Prediction studies in medicine and psychiatry have changed markedly since the advent of machine learning (ML) and artificial intelligence (AI). Contemporary approaches usually show better performance compared with traditional parametric analyses (which make assumptions that may not hold in clinical practice). However, even widely used ML models that include decision tree analysis (e.g., classification and regression tree) and receiver operating characteristic curve (ROC) analysis are susceptible to overfitting or poor generalisability to other samples [28]. Many groups have adopted ensemble ML models such as Boosting alongside quality assurance metrics that are more reliable when there is an imbalanced sample, such as the Matthews correlation coefficient [29]. Boosting is a method that is used widely to analyse imbalanced samples (where predicted case numbers comprise only a small proportion of the total study population and where a single ML model often makes prediction errors because of issues with the training sample). Boosting tries to overcome these weaknesses by training multiple ML models sequentially to improve the accuracy of the overall system. The findings from Boosting can then be used to generate a ROC curve to estimate the overall accuracy of the model, whilst the MCC is viewed as an easy to interpret and superior measure of the quality of binary classifications. It may be useful to explore how an ensemble ML model performs when assessing onset of BD‐I or BD‐II as this approach appears to have the advantage of reducing bias in the classification and is often applied in models that predict rare diseases, cancers and also suicides (where the outcome is relatively rare and there is a significant risk of mis‐classifying cases as non‐cases) [30].

Accordingly, this study explores the extent to which a combination of FH, PRS, and clinical phenomenology (e.g., self‐rated symptoms or sub‐threshold syndromes), applied within the contemporary Boosting framework, can predict BD onset in a prospective cohort of adolescents and young adults in the peak age range for onset of mood and psychotic disorders.

## 2 Methods

### 2.1 Study Overview

This study is part of a programme of research about the health and well‐being of the Brisbane Longitudinal Twin Study (BLTS) cohort. It received ethical approval from the Human Research Ethics Committee at the Queensland Berghofer Institute of Medical Research and the University of Sydney (references: EC00278 and P1212).

This report follows Strengthening the Reporting of Observational Studies in Epidemiology (STROBE) guidelines (Appendix 1 provides the STROBE checklist). Here, we provide a summary of eligibility criteria, and descriptions of clinical assessments and PRS estimates relevant to these analyses. Additional details about the protocol, procedures and data collection processes are provided in online materials (Appendix 2). Further information and other research findings are available in recent cohort publications (see Appendix 2).

#### 2.1.1 Participants

The cohort for this study was derived from the BLTS database, which includes encrypted, de‐identified data from a prospective, longitudinal, community‐based cohort of twins and non‐twin siblings who were recruited from Brisbane, Queensland, via media appeals and word of mouth from 1992 onwards. Ethnically, the cohort reflects the population structure of the greater Brisbane area at the time of recruitment, with most participants of European ancestry and minorities of predominantly Asian ancestry. Individuals were eligible to join the cohort from age 12 onwards (with written parental consent), and mental health assessments were introduced from age ~ 15 onwards.

This study focuses on data collected between the ages of ~15–25 years, particularly the 19Up and 25Up follow‐ups that included detailed self‐rating and interview‐based assessments of mental health. Due to the nesting of the data collection within a longitudinal framework, individuals who missed a follow‐up could be invited to participate at the next wave, and findings from recent cross‐sectional assessments can be linked to those from earlier waves [31].

For the prediction analyses, we extracted de‐identified individual data according to the following inclusion criteria:

- the participant had completed all self‐ratings of psychopathology at a follow‐up undertaken between ages 15–19;
- the Composite International Diagnostic Interview (CIDI) [32] and family history (FH) of major mental disorders assessment were available from the 19Up or 25Up follow‐up;
- age at onset of self‐rated psychopathology (e.g., sub‐threshold syndromes) and/or of the first full‐threshold CIDI episode was recorded (or could be estimated from information available).

Individuals were excluded if no PRS estimates were available or PRS estimates did not meet quality control standards. Likewise, we excluded individuals if the age at completion of the CIDI assessment and/or estimated age at onset of any full‐threshold syndrome *preceded* the age at completion of the symptom self‐ratings, and/or if insufficient data were recorded, and/or ages at onset could not be estimated or specific age data were missing.

Using these criteria, we identified that 1473 individuals (out of 1815 potential participants) met all the eligibility criteria (see supplementary Table 1S for the comparison of baseline characteristics in the 1473 cohort members who were included versus the 342 who were excluded).

### 2.2 Clinical Assessments

#### 2.2.1 Demography

Key characteristics were recorded (see Table 1).

**TABLE 1:** Key characteristics of the study cohort (*n* = 1473).

Characteristic	Sample (*N* = 1473)
Mean Age in years (SD)	26.3 (4.4)
	**Number (%)**
Females	866 (59%)
European ancestry	1370 (93%)
Educational Level: Junior or Senior School only	267 (18%)
Full‐Time Employment	869 (59%)
Civil Status: Single	998 (55%)
Zygosity	
Monozygotic Twins	427 (29%)
Dizygotic Twins	530 (36%)
Non‐Twin Siblings	516 (35%)
Full‐Threshold Mood or Psychotic Disorder	409 (29%)
Bipolar Disorder	
Hypomania	74 (5%)
Mania	30 (2%)
Self‐Reported Help‐Seeking for a Mental Health Problem	388 (26%)

#### 2.2.2 CIDI Caseness

We applied established algorithms to CIDI assessment data to determine the presence or absence of a range of DSM‐IV disorders and their age at onset (Kessler et al. 2012 [32]). We extracted data regarding major depressive, hypomanic, manic or psychotic syndromes that met CIDI criteria for caseness (Appendix 2).

#### 2.2.3 Self‐Rated Psychopathology

Details about the self‐ratings used in this study are provided in Appendix 2 and in our previous publications. In brief, individuals completed self‐ratings of 23 items that may indicate the presence of hypomanic/manic (HMMSx), depressive (DepSx), or psychotic symptoms (PSx) and can be used to identify whether any clusters of mood or psychotic symptoms meet the criteria for a sub‐threshold mood and/or psychotic syndrome, referred to as Hypomanic‐like (HMLE), Depressive‐like (DLE), or Psychotic‐Like Experiences (PLE). The rating scales employed were chosen because they have been widely used to evaluate psychopathology experienced by young people in community and clinical settings and because the ratings can be examined from several perspectives, including prediction of transition to caseness in studies of BD, depressive, and psychotic disorders (Appendix 2 provides references).

#### 2.2.4 Family History (FH) of Mood and Psychotic Disorders

The presence or absence of mood and psychotic disorders in 1st degree family members was identified using an online assessment based on the Family History Screen [33]. Multigenerational‐FH and FH of mood and psychotic disorders were identified from self‐report data (Appendix 3).

#### 2.2.5 Help‐Seeking Behaviour

We extracted information about help‐seeking from self‐report of mental health problems, engagement in help‐seeking and/or recommendations for treatment of a mental health problem as recorded via the Headspace National Telephone Survey [34].

### 2.3 Estimating Polygenic Risk Scores

Genotyping and quality control procedures for the BLTS cohort are described in detail elsewhere [35]. The PRS for BD, MDD, SCHZ and NEU used in this analysis were constructed from the data releases from the Psychiatric Genomics Consortium and are based on well‐powered multi‐national GWAS conducted in adult populations (for full explanation see https://pgc.unc.edu/for‐researchers). It should be noted that given the geographic distribution of the discovery samples used in the GWAS, the genetic data available to each consortium are known to be more representative of Caucasian (European ancestry) populations.

As noted in Appendix 2, a PRS provides a statistical estimate of an individual's common genetic liability to a trait and is calculated as a weighted sum of their individual load of single nucleotide polymorphisms (SNPs) associated with that trait, weighted by an effect size derived from summary statistic GWAS data [36]. In our analyses the PRS are represented by the SBayesR estimates for NEU, MDD, BD and SCHZ. In brief, the SBayesR algorithm is preferred to many other PRS estimations as it offers a non‐parametric Bayesian method that gives the highest prediction accuracy for psychiatric disorders in independent samples [35]. The individual PRS for NEU, MDD, BD and SCZ are expressed as single value estimates (standardised scores which can be negative or positive), with higher positive scores indicating an individual has a higher genetic liability for the trait [36]. In addition, we wished to examine the shared effects of these PRS as well as their individual contribution to the prediction models. Therefore, we undertook a principal component analysis (PCA) of the four PRS to derive statistically reliable and clinically interpretable PRS dimensions for further details, see Scott et al. [15]. The PCA identified two dimensions accounting for 70% of the explained variance; the first dimension, which we labelled MDD‐NEU, accounted for 35.7% of the explained variance; the second, which we labelled BD‐SCHZ, accounted for 34.3% (Appendix 3). These two dimensions were employed in some of the statistical analyses described below.

### 2.4 Statistical Analyses

Predictive analyses were undertaken using the ‘Generalised Boosted Regression Modelling’ (gbm) R package (https://CRAN.R‐project.org/package=gbm, version 2.2) and the processes are outlined below (additional details are provided in Appendix 2).

The Boosting model is based on classification and regression decision trees (CARTs) and the ensemble model was constructed by sequentially combining multiple “weak” decision trees generated from the familial, genetic and clinical risk factors for BD included in the predictive analysis. It should be noted that the samples utilised at each stage of the Boosting analysis were not uniformly drawn from the same population; instead, cases that were incorrectly predicted in a prior step received increased weight in the subsequent step [37]. As such, the Boosting algorithms enhanced predictions of BD onset iteratively, helping to reduce the high bias often associated with shallow decision trees and logistic regression models [38]. To enhance the robustness and accuracy of the final Boosting models, the gbm package selects different subsamples of the BLTS cohort for model training (60% of the sample with 10‐fold cross‐validation), validation (20%) and test sets (20%) for each analysis [39].

Here, we examined the incremental improvements in the BD prediction model by the stepwise introduction of risk factors (see below). The gbm package allowed reporting of the relative influence (in some packages referred to as feature importance or FI) of each variable within each Boosting model (Appendix 2), whilst the overall accuracy of the prediction model was evaluated by estimating the area under the curve (AUC) for the ROC (AUROC with 95% confidence intervals; 95% CI) and the quality of the binary classification was estimated using the Matthews correlation coefficient (MCC).

Zou et al. [40] suggest that an AUROC estimate of at least 0.8 is required to demonstrate clinical utility as well research significance for a medical screening or diagnostic test. Similarly, Namh [41] notes that an AUROC > 0.8 is viewed as “good” and ≥ 0.9 as “very good”, and acceptable screening tools typically have an AUROC ≥ 0.85. The MCC is a measure of the correlation between the predicted and true class labels and is relatively easy to interpret. The MCC considers all four possible outcomes of a binary classification (true and false positives; true and false negatives) and is therefore more balanced than other metrics such as accuracy or precision [29]. This makes it useful for studies such as prediction of BD onset where the classes are imbalanced, and the cost of false positives and false negatives is quite different (i.e., not identifying true cases would have worse consequences than mistakenly identifying a healthy person as a potential case). The MCC estimate ranges from −1 to 1, where 1 is a perfect classification, 0 is no better than chance, and − 1 is a completely incorrect classification. An MCC of 0.3 to 0.5 suggests a “moderately good” binary classification model, while an MCC ≥ 0.5 is a “good” model.

As this is an exploratory study, we repeated the Boosting analysis several times to enable determination of the optimal combination of FH, PRS or clinical features (separately and then in combination) that classified individuals according to (i) BD (subtypes BD‐I and BD‐II); (ii) Manic caseness; and (iii) Hypomanic caseness. Previous research suggests that the models for prediction of Mania and Hypomania may differ, so we planned a subsidiary analysis to examine whether help‐seeking status influences the accuracy of the classifications of BD subtypes (see below), as individuals experiencing Hypomania may be less likely to seek help than those experiencing Mania.

The analyses proceeded in the following steps:

Step 1: Baseline Variables: before undertaking the predictive analyses, we ran a Boosting model incorporating variables that are known to influence case–control classifications in BD. These included age, sex, twin status, etc. (for further details see Appendix 2 and Scott et al. [15]). This baseline classification was used to generate a single composite predictor variable (hereafter referred to as ASAFT to reflect the names of the variables) that was then included in each of the analyses of different combinations of putative FH, PRS, and clinical risk factors.

Step 2: The next step considered whether FH and PRS estimates improved upon the baseline classification. We tested different representations of these risk factors e.g., for FH we ran models that explored not only the presence or absence of FH BD, FH Psychosis and FH MDD, but also a FH of both mood and psychotic disorders, (i.e., more than one severe mental disorder) and the presence of a multi‐generational FH. Likewise for genetic vulnerability, we examined the PRS for BD, SCHZ, MDD, and NEU, and the two PCA‐derived PRS dimensions (BD‐SCHZ, MDD‐NEU).

Step 3: In this step we considered whether including different measures of antecedent psychopathology improved the classification of BD caseness obtained previously. We ran models that included the presence or absence of categorically defined risk syndromes (i.e., DLE, PLE and/or HMLE) prior to a full‐threshold mood or psychotic episode and then examined the predictive utility of number of depressive (DepSx), psychotic (PSx) or hypomanic (HMMSx) symptoms (symptom scores were Z‐transformed).

Step 4: We then examined whether including help‐seeking status in the classification model may shed light on previous differences in predictors of mania or hypomania in clinical versus non‐clinical samples.

The best model for each classification was selected according to the following criteria: the model demonstrated the highest AUROC with the ROC for classification of BD cases exceeding that of non‐cases and the model had a higher MCC than any similar models (undertaken at the same step). These predictors were then included in a combined model and the AUROC and MCC were examined alongside the estimated relative influence of each risk factor included in this final BD classification model. We then examined whether these features were able to predict Manic caseness or Hypomanic caseness to the same extent.

## 3 Results

### 3.1 Sample Description

As shown in Table 1, the cohort comprised 1473 participants (female = 866; 59%) with a mean age of 26.3 (SD = 4.4). Over 90% (*n* = 1370) were of genetically inferred European ancestry; 55% were single and 59% were in full‐time employment. About 29% (*n* = 409) met criteria for a full‐threshold CIDI mood or psychotic disorder; 116 (28% of these 409 cases) met criteria for > 1 CIDI disorder (supplementary Figure 1S). The median age at first onset of a full‐threshold CIDI diagnosis was ~20 years (Interquartile Range: 18–23). One‐hundred‐and‐four individuals met criteria for BD (7%), with 30 (2%) experiencing at least one manic episode. Three‐hundred‐and‐eighty‐eight members self‐reported help‐seeking, of which 63% (*n* = 245/388) met CIDI criteria for a full‐threshold mood or psychotic disorder.

**FIGURE 1:**
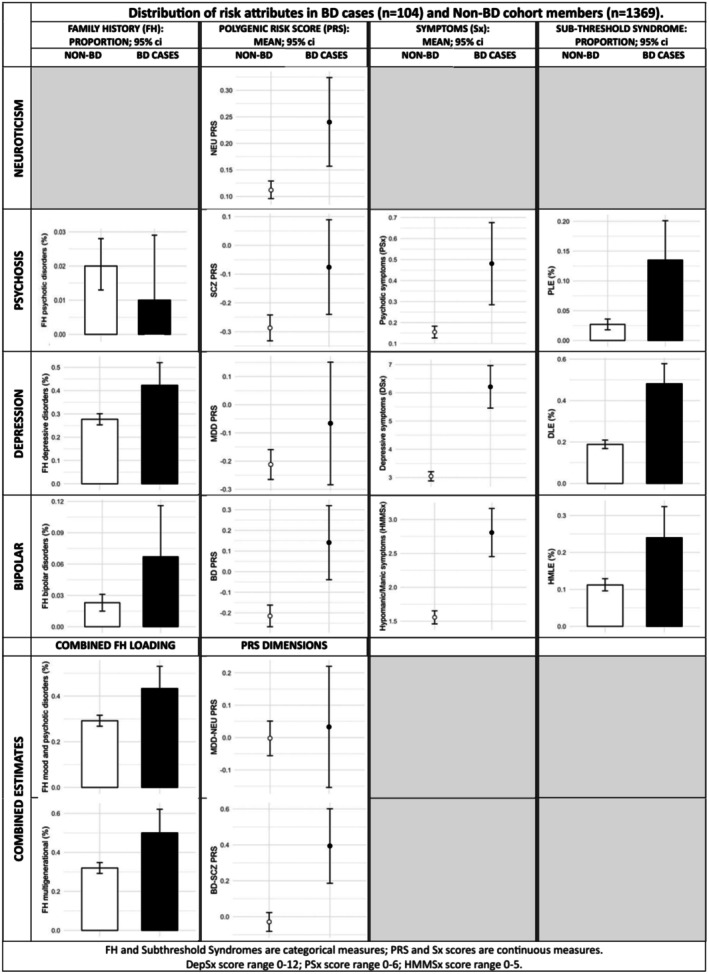
Distribution of risk attributes in BD cases (*n* = 104) and Non‐BD cohort members (*n* = 1369).

Figure 1 summarises the distribution of risk factors in BD cases (*n* = 104) compared with other participants (*n* = 1369). The magnitude of familial, genetic and psychopathology loadings is higher in BD cases except for FH of psychotic disorders, which is higher in the Non‐BD group (which includes healthy controls but also individuals who have other CIDI diagnoses including psychotic and depressive disorders).

### 3.2 Boosting Analyses

The preliminary Boosting model identified that baseline variables (ASAFT) gave an overall AUROC of 55% (95% CI: 49%, 61%; MCC = 0.11). Table 2 reports key findings for the optimal model at each step (metrics for sub‐optimal classifications are shown in Table S2).

**TABLE 2:** Optimal boosting models for predicting onset of BD utilising different risk attributes.

Model*	ROC for Classification (%)	Relative Influence**	
Relative influence			
OVERALL	61.6	MULTI‐GEN FH	51.3
BD	62.7	FH BD	20.62
No BD	60.5	FH PSY	5.19
		FH MDD	3.1
Polygenic Risk Scores (PRS)			
OVERALL	65.3	BD‐SCHIZ	84.2
BD	67.1	MDD‐NEU	12.51
No BD	63.5		
FH + PRS			
OVERALL	71.9	BD‐SCHIZ	62.45
BD	72.2	FH‐BD	17.91
No BD	71.6	FH‐MDD	8.74
		MDD‐NEU	7.64
		MULTI‐GEN FH	3.26
Clinical Risk Syndromes/Symptoms (Sx)			
OVERALL	67.9	DepSx	67.16
BD	72.3	PSx	27.79
No BD	67.3	HMMSx	5.08
FH + PRS + Sx			
OVERALL	85.1	DepSx	49.57
BD	86.7	PSx	22.78
No BD	82.8	BD_SCHIZ	17.01
		HMMSx	6.91
		FH‐BD	3.73

*Note:* ROC: Receiver Operating Characteristic (see text for 95% confidence intervals); MULTI‐GEN FH: multi‐generational FH; BD‐SCHIZ and MDD‐NEU refer to PRS Dimensions (see text for details).

* All prediction models include baseline variables such as age, sex, twin status, etc. (i.e., ASAFT; see text for details).

** Relative Influence also known as feature importance metric.

The best performing Boosting model for the predictive utility of FH attributes gave an overall AUROC of 61.6% (95% CI: 56%, 68%; MCC = 0.25). The variables with the greatest relative influence (in rank order) were: multigenerational‐FH; FH‐BD; FH‐Psychosis; and FH‐MDD.

When the Boosting classification included different PRS profiles, the best performing model included the PRS dimensions. This model had a slightly better classification rate than the FH model, with an overall AUROC of 65.3% (95% CI: 59%, 72%; MCC = 0.26) and an AUROC for correctly identifying BD cases of 67.1%. The relative influence of the BD‐SCHIZ PRS dimension was 7× greater than the MDD‐NEU PRS dimension.

Combining FH and PRS variables into a single model demonstrated a further improvement in the prediction of BD caseness (overall AUROC 71.9%; 95% CI: 66%, 78%; MCC = 0.31); the features with highest importance were the PRS dimension of BD‐SCHIZ and FH‐BD.

When Boosting explored the predictive utility of different patterns of psychopathology (symptoms or subthreshold syndromes; Figure 1), the model incorporating symptom measures (rather than subthreshold syndromes) had a higher overall AUROC (67.9%; 95% CI: 63%, 74%; MCC = 0.28), correctly identifying 72.3% of BD cases. Interestingly, DepSx were ranked as having the highest feature importance followed by PSx, then HMMSx.

As shown in Table 2, the best performing Boosting classification that included FH, PRS and clinical variables had an overall AUROC of 85.1% (95% CI: 80%, 88%) and correctly identified 86.7% of BD cases (MCC = 0.46). The variables with greatest relative influence were: DepSx, PSx and the BD‐SCHIZ dimension, whilst HMMSx and FH‐BD showed lower feature importance.

**FIGURE 2:**
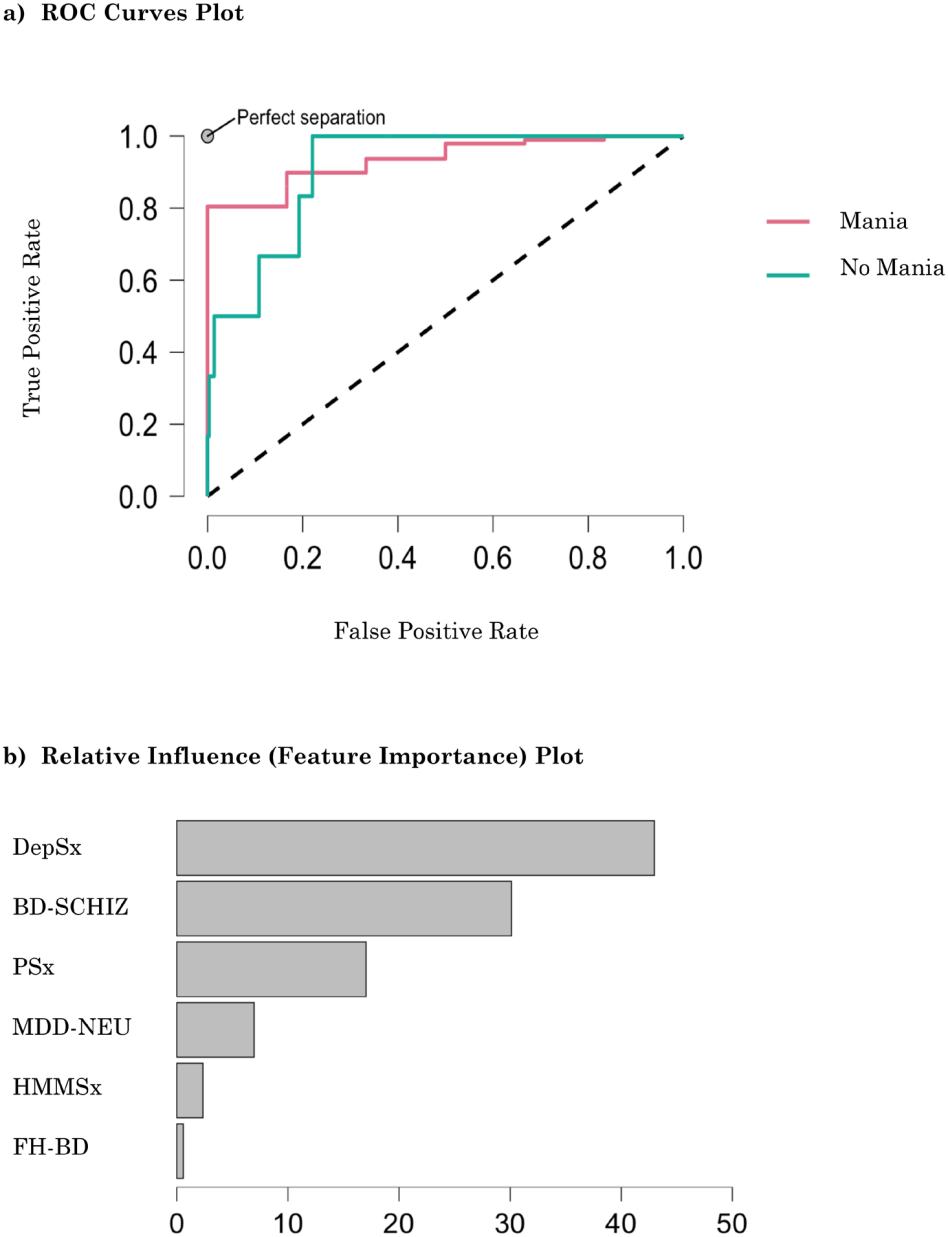
Boosting classification of Mania (*n* = 30) ROC Curves Plot Mania.

Finally, as shown in Figure 2, the optimal model for predicting BD caseness (BD‐I and BD‐II) was particularly useful for predicting Manic caseness. The AUROC was 89% (95% CI: 81%, 97%) for identifying cases with at least one episode of mania (MCC = 0.42). The rank order for feature importance was: DepSx, BD‐SCHIZ PRS dimension, PSx, MDD‐NEU PRS dimension, then HMMSx and FH‐BD. Adding help‐seeking status into the model predicting Mania had minimal impact on the performance of the model. In contrast, the overall AUROC for the prediction of hypomania improved from 68% (95% CI: 61%, 75%; MCC = 0.18) to 79% (95% CI: 73%, 85%) when help‐seeking status was included. The MCC for the latter model was 0.26 and the rank order for features of most importance was: DepSx (37.3); HMMSx (22.8); MDD‐NEU (20.6); BD‐SCZ (10.2); and help‐seeking status (7.3).

## 4 Discussion

The impetus for this proof‐of‐concept study was four‐fold. First, we wanted to examine whether we could use information about risk factors derived from different data modalities (self‐report, observer recordings, molecular genetics) to identify an optimal model for the prediction of BD onset that met the *minimum criteria for clinical utility* (i.e., AUROC 80%–90%). Second, we wished to explore whether the optimal prediction model was *equally accurate for predicting manic and hypomanic onsets*. Third, we decided to investigate any additional gains from using *different approaches to measuring risk attributes* (e.g., disorder‐specific PRS vs. PRS dimensions, etc.). or to identifying sub‐populations (e.g., help‐seeking status). Fourth, we wanted to highlight the need to employ predictive models that consider the fact that individuals who develop a specific mental health condition, such as BD, are typically in the minority, i.e., *we sought a model that prioritised the prediction of caseness*. As noted by Genetili et al. this is especially important when studying real‐world datasets, as these are inherently imbalanced, and if extreme imbalances exist (where cases represent only 1%–3% of the study population), this frequently leads to predictions biased toward the majority class (i.e., the model will always be better at predicting the healthy comparator or ‘non‐case’ class) [42].

Our main findings were that our optimal Boosting model accurately classified over 85% of BD cases and that a combination of mood and psychotic symptoms, PRS dimensions and FH of BD were the most important risk factors in predicting caseness. At a superficial level, the prediction model appears to lack new insights into risk factors associated with future onset of BD. However, a more detailed review of the findings demonstrates that the relative importance of some risk factors differs from the emphasis given to those factors in existing research and/or a more nuanced approach to operationalisation of risk attributes may enhance the accuracy of the prediction model (and increase the likelihood the overall model meets the criteria for clinical utility)., For example, depressive symptoms (DepSx) and psychotic symptoms (PSx) were ranked as more important than hypomanic/manic symptoms (HMMSx). Although this might seem counter‐intuitive, it is in keeping with other studies on the early phenomenology of BD. Depressive phenomena are well‐established antecedents of BD, but contemporary studies and reviews increasingly emphasise the importance of ‘activation’ as a core phenotype of BD [43, 44]. Activation is primarily associated with changes in rest‐activity rhythms and subjective energy, and we know that these symptoms are frequently included in depression rating scales in the form of *decreased* activation (measured as low subjective energy and psychomotor retardation). Although items regarding *increased* activation (i.e., increased energy, psychomotor excitation) are included in some hypo/mania screening tools, many scales place less emphasis on these features compared with mood ratings of elation and instability. Against this phenomenological background, we were unsurprised about the relative importance of DepSx load. Likewise, we have shown elsewhere that symptoms labelled as psychotic, such as paranoia, are not specific to individuals at clinical high risk of psychosis and are also common in those at high‐risk of BD, especially mania [17, 31]. Whilst the current study did not itemise individual symptoms, we suggest that the finding that symptoms, rather than antecedent sub‐threshold syndromes, were more useful in predicting future onset of BD is important. Being able to identify individual symptoms or symptom clusters that may represent important clinical phenotypes (irrespective of items being included in rating scales of depression, hypomania, psychosis, etc.). offers a more refined approach than employing categorical measures of sub‐threshold syndromes (which typically focus on the number rather than the nature of any symptoms). A further argument in support of a symptom‐level approach can be inferred from our finding that our optimal prediction model was not only less accurate in predicting hypomania (NB: even with help‐seeking included, the overall accuracy of the model was < 80%) but the models also differed in the relative importance of psychotic versus hypomanic symptoms (and of the MDD‐NEU versus the BD‐SCHIZ PRS dimensions). An ongoing concern for researchers in the field is that many screening tools for BD struggle to differentiate phenomenology associated with hypomania from that associated with different temperamental and personality styles in youth (and in some instances from ADHD). Investigating symptom profiles in more detail (e.g., activation versus mood lability) might shed light on whether different risk pathways exist for mania and hypomania [45]. Also, it would allow investigation of whether the PRS findings (for mania versus hypomania) are genuinely accounted for by different pathways, or for example, are the PRS differences better explained by previously identified associations between PRS and clinical severity or help‐seeking status [15, 18]. Such research would not only have implications for BD screening but also potentially inform future discussions about the optimal classification of BD subtypes according to core (or more specific) phenomena.

Other relevant study findings were that the stepwise inclusion of different markers of risk associated with FH and PRS made small but measurable improvements in the overall accuracy of the preliminary prediction model (based on sample characteristics alone). Also, the proportion of phenotypic variance explained by the inclusion of PRS (~5%) matches findings reported in studies of BD [8, 22, 46] and other mental disorders [47]. Our study demonstrated that PRS derived from a PCA‐derived dimensional model improved the accuracy of the prediction compared with the univariate (disorder‐specific) PRS. This approach has not yet been used in other studies of BD onset, but other researchers have employed this strategy in child and adolescent research [20], and Grimes and colleagues [48] found that the use of multi‐trait PRS improved their prediction model for onset of depression in youth. There is a lack of agreement regarding the utility of PRS in predicting BD‐I and BD‐II, as some research, such as offspring studies, report that PRS BD is equally useful in identifying mania and hypomania [8], whilst other population‐based studies found that the current PRS for BD lacked sufficient power to separate individuals into clinically meaningful BD categories [18]. These inconsistent findings may partly be explained by differences in study design, sampling, and definitions of clinical high‐risk phenotypes. However, there are broader issues facing research using high dimensional genetic data, as emerging evidence suggests that PRS for the same disorder, but estimated from different discovery GWAS, may lack stability; for example ~20% of individuals identified as high risk with one PRS for posttraumatic stress disorder (PTSD) were identified as high risk with another PRS for PTSD (NB): the stability estimate for PRS MDD was about 50% [49]. This is an evolving field, and it is hoped that newer PRS estimates will prove to be more stable, which will be important if they are to be employed in health services for prognostication or clinical decision making. However, it is yet to be determined whether dimensional (multi‐trait) PRS will be more useful than univariate PRS [50], or whether alternatives approaches, such as estimating Differential Diagnosis PRS (“DDx PRS”) might be a better option for predicting divergent illness trajectories [51].

This proof‐of‐concept study of BLTS has strengths and weaknesses. There is evidence that our study participants are comparable to other adolescent and young adult populations described elsewhere according to prevalence rates for risk factors, CIDI diagnoses and comorbidities [52]. This representativeness is further reflected in the reported rates of mania and hypomania identified in this community‐residing cohort. However, even though the study specifically utilised analytic techniques to cope with the fact that BD cases were in a minority, we are aware that the model for the prediction of mania is based on 30 individuals. Furthermore, it would be incorrect to assume that BLTS cohort (a third of which comprised monozygotic twins) is representative of all other prospective community‐based studies in this field. The BLTS cohort cannot be regarded as a random sample of the general population and so twinning and relatedness were included as potential confounders in all the planned analyses. A unique strength of the BLTS is that prospective longitudinal clinical ratings are available as well as FH and PRS data. However, we did not have independent corroboration of FH and, like many PRS studies, the PRS estimates are derived mainly from GWAS in individuals of European ancestry. The latter undermines the potential utility of including PRS in prediction models in some geographic locations. In addition, the cost of PRS testing (about 20USD per person) may limit opportunities for research or clinical use of this approach. It should be noted that, the specific PRS dimensions we used (BD‐SCHIZ and MDD‐NEU), although similar to other multi‐state dimensions, have not yet been employed elsewhere. It is always important to confirm findings from ML models, but the issues highlighted here mean that the predictive models we have identified need replication in independent samples and that more diverse data are needed to ensure generalisability.

## 5 Conclusions

To date, predicting the onset of BD in young adults has primarily been achieved by estimating the individual level of risk associated with a combination of factors representing FH and antecedent psychopathology. The recent inclusion of PRS has also proved useful for risk stratification in research settings. However, previous research has failed to explore the best operationalization of the different risk factors (FH‐BD versus multigenerational FH; symptoms versus syndromes; disorder‐specific versus dimensional PRS) and the overall accuracy of most predictive models has failed to reach the threshold for clinical utility (AUCs = 80%–90%). Furthermore, those models mostly used comparator groups that excluded other cases of mental disorders. Our study examined the prediction of BD versus other cohort members (i.e., healthy individuals, those with psychosis, those with MDD etc.). This study contributes to this field of research as it identifies which specific combinations of clinical and FH information (i.e., routinely available in clinical case notes) and PRS data (obtained via a blood test costing ~$20 USD) provided the highest prediction accuracy according to a statistical model that considered that BD onset only occurs in a small minority of most populations. The findings highlight that it is not just what you chose to measure (as risk attributes), but how you measure it (operationalisation) and how you analyse potential predictors in imbalanced samples to ensure the model will always be better at predicting the cases (rather than the healthy comparator or ‘non‐case’ class). The insights gained from this proof‐of‐concept study can be used to inform future research in this field, and potentially applications to early intervention mental health clinics.

## Funding

This work was supported by the National Health and Medical Research Council, Australia (1031119). National Health and Medical Research Council, Australia (1049911). National Health and Medical Research Council, Australia (APP10499110). National Institute of Health, USA (R00DA023549).

## Conflicts of Interest

S.E.M. is supported by an NHMRC SRF APP1103623. J.J.C. is supported by an NMHRC Emerging Leadership Fellowship (GNT2008197). B.L.M. is supported by an NMHRC Emerging Leadership Fellowship (GNT2017176). N.G.M. is supported by an NMHRC Leadership Fellowship (1172990). J.S. is a visiting professor at the Brain and Mind Centre (Sydney) and at Universite de Paris, the Norwegian University of Science and Technology (Trondheim) and is a “Science without Borders” fellow (Brazil). She has received grant funding from the UK Medical Research Council and from the UK NIHR Research for Patient Benefit programme; she declares no financial or other conflicts of interest in relation to the topics addressed in this article. I.B.H. is the Co‐Director, Health and Policy at the Brain and Mind Centre (BMC), University of Sydney. The BMC operates an early intervention youth service at Camperdown under contract to headspace. He is the Chief Scientific Advisor to, and a 3.2% equity shareholder in, InnoWell Pty Ltd. InnoWell was formed by the University of Sydney (45% equity) and PwC, Australia (45% equity) to deliver the $30 M Australian Government‐funded Project Synergy (2017–20; a three‐year program for the transformation of mental health services) and to lead transformation of mental health services internationally using innovative technologies.

## Supporting information


**Appendix 1** STROBE checklist.
Appendix 2: Overview of BLTS & further details of assessment tools, analysis and additional references.
Appendix 3: Supplementary Tables and Figures.
**Table S1:** Comparison of cases included in the study cohort (*n* = 1473) with excluded cases (*n* = 342).
**Figure S1:** Diagrammatic representation of CIDI diagnoses in sample of 1473.
**Table S2:** Sub‐optimal models generated from boosting analyses of risk attributes.

## Data Availability

The data that support the findings of this study were made available to authors via the BLTS research committee (that approved the cohort study). The authors confirm that the summary data for all variables supporting the findings of this study are included within the article and its Supporting Information. The raw data are being used at the lead research centres and form part of an ongoing programme of research and data are only made available upon written request to the BLTS research committee. Data are not publicly available due to confidentiality restrictions and because research participants did not give permission for dissemination beyond the BLTS research team. As such, outside investigators seeking access to data from this project would need to contact QMIR with a view to submitting a detailed proposal outlining study aims, hypotheses, variables or constructs, and the intended analytic approach.

## References

[1] J. Kalman , L. Olde Loohuis , A. Vreeker , et al., “Characterisation of Age and Polarity at Onset in Bipolar Disorder,” British Journal of Psychiatry 219, no. 6 (2021): 659–669. 10.1192/bjp.2021.102 PMC8636611 35048876

[2] J. McGrath , A. Al‐Hamzawi , J. Alonso , et al., “Age of Onset and Cumulative Risk of Mental Disorders: A Cross‐National Analysis of Population Surveys From 29 Countries,” Lancet Psychiatry 10, no. 9 (2023): 668–681. 37531964 10.1016/S2215-0366(23)00193-1 PMC10529120

[3] D. Hafeman , J. Merranko , T. Goldstein , et al., “Assessment of a Person‐Level Risk Calculator to Predict New‐Onset Bipolar Spectrum Disorder in Youth at Familial Risk,” JAMA Psychiatry 74, no. 8 (2017): 841–847. 28678992 10.1001/jamapsychiatry.2017.1763 PMC5710639

[4] J. Carpenter , J. Scott , F. Iorfino , et al., “Predicting the Emergence of Full‐Threshold Bipolar I, Bipolar II and Psychotic Disorders in Young People Presenting to Early Intervention Mental Health Services,” Psychological Medicine 52, no. 10 (2022): 1990–2000. 33121545 10.1017/S0033291720003840

[5] R. Uher , B. Pavlova , J. Radua , et al., “Transdiagnostic Risk of Mental Disorders in Offspring of Affected Parents: A Meta‐Analysis of Family High‐Risk and Registry Studies,” World Psychiatry 22, no. 3 (2023): 433–448. 37713573 10.1002/wps.21147 PMC10503921

[6] K. Dean , H. Stevens , P. Mortensen , R. Murray , E. Walsh , and C. Pedersen , “Full Spectrum of Psychiatric Outcomes Among Offspring With Parental History of Mental Disorder,” Archives of General Psychiatry 67, no. 8 (2010): 822–829. 20679590 10.1001/archgenpsychiatry.2010.86

[7] A. Duffy , S. Doucette , U. Lewitzka , M. Alda , T. Hajek , and P. Grof , “Findings From Bipolar Offspring Studies: Methodology Matters,” Early Intervention in Psychiatry 5, no. 3 (2011): 181–191. 21718461 10.1111/j.1751-7893.2011.00276.x

[8] B. Birmaher , D. Hafeman , J. Merranko , et al., “Role of Polygenic Risk Score in the Familial Transmission of Bipolar Disorder in Youth,” JAMA Psychiatry 79, no. 2 (2022): 160–168. 34935868 10.1001/jamapsychiatry.2021.3700 PMC8696688

[9] S. Mistry , J. Harrison , D. Smith , V. Escott‐Price , and S. Zammit , “The Use of Polygenic Risk Scores to Identify Phenotypes Associated With Genetic Risk of Bipolar Disorder and Depression: A Systematic Review,” Journal of Affective Disorders 234 (2018): 148–155. 29529547 10.1016/j.jad.2018.02.005

[10] X. Jiang , C. Zai , M. Dimick , et al., “Psychiatric Polygenic Risk Scores Across Youth With Bipolar Disorder, Youth at High Risk for Bipolar Disorder, and Controls,” Journal of the American Academy of Child and Adolescent Psychiatry 63, no. 11 (2024): 1149–1157. 38340895 10.1016/j.jaac.2023.12.009

[11] D. Hafeman , R. Uher , J. Merranko , et al., “Person‐Level Contributions of Bipolar Polygenic Risk Score to the Prediction of New‐Onset Bipolar Disorder in At‐Risk Offspring,” Journal of Affective Disorders 368 (2025): 359–365. 39299598 10.1016/j.jad.2024.09.107 PMC11869166

[12] K. Musliner , M. Krebs , C. Albinana , et al., “Polygenic Risk and Progression to Bipolar or Psychotic Disorders Among Individuals Diagnosed With Unipolar Depression in Early Life,” American Journal of Psychiatry 177, no. 10 (2020): 936–943. 32660297 10.1176/appi.ajp.2020.19111195

[13] V. Rodriguez , L. Alameda , D. Quattrone , et al., “Use of Multiple Polygenic Risk Scores for Distinguishing Schizophrenia‐Spectrum Disorder and Affective Psychosis Categories in a First‐Episode Sample; the EU‐GEI Study,” Psychological Medicine 53, no. 8 (2023): 3396–3405. 35076361 10.1017/S0033291721005456 PMC10277719

[14] A. Zwicker , J. Fullerton , N. Mullins , et al., “Polygenic Scores and Onset of Major Mood or Psychotic Disorders Among Offspring of Affected Parents,” American Journal of Psychiatry 180, no. 4 (2023): 285–293. 36856707 10.1176/appi.ajp.20220476

[15] J. Scott , J. J. Crouse , S. Medland , et al., “Polygenic Risk Scores and the Prediction of Onset of Mood and Psychotic Disorders in Adolescents and Young Adults,” Early Intervention in Psychiatry 18, no. 6 (2024): 397–405. 37787636 10.1111/eip.13472 PMC11100301

[16] K. Yao , T. van der Veen , J. Thygesen , N. Bass , and A. McQuillin , “Multiple Psychiatric Polygenic Risk Scores Predict Associations Between Childhood Adversity and Bipolar Disorder,” Journal of Affective Disorders 15, no. 341 (2023): 137–146. 10.1016/j.jad.2023.08.116 37643680

[17] N. Samani , E. Beeston , C. Greengrass , et al., “Polygenic Risk Score Adds to a Clinical Risk Score in the Prediction of Cardiovascular Disease in a Clinical Setting,” European Heart Journal 45, no. 34 (2024): 3152–3160. 38848106 10.1093/eurheartj/ehae342 PMC11379490

[18] K. O'Connell , M. Koromina , T. van der Veen , et al., “Genomics Yields Biological and Phenotypic Insights Into Bipolar Disorder,” Nature 639, no. 8056 (2025): 968–975. 39843750 10.1038/s41586-024-08468-9 PMC12163093

[19] P. Lichtenstein , B. Yip , C. Bjork , et al., “Common Genetic Determinants of Schizophrenia and Bipolar Disorder in Swedish Families: A Population‐Based Study,” Lancet 373, no. 9659 (2009): 234–239. 19150704 10.1016/S0140-6736(09)60072-6 PMC3879718

[20] A. Neumann , A. Jolicoeur‐Martineau , E. Szekely , et al., “Combined Polygenic Risk Scores of Different Psychiatric Traits Predict General and Specific Psychopathology in Childhood,” Journal of Child Psychology and Psychiatry, and Allied Disciplines 63, no. 6 (2022): 636–645. 34389974 10.1111/jcpp.13501 PMC9291767

[21] A. Rodrigue , S. Mathias , E. Knowles , et al., “Specificity of Psychiatric Polygenic Risk Scores and Their Effects on Associated Risk Phenotypes,” Biol Psychiatry Glob Open Sci 3, no. 3 (2022): 519–529. 37519455 10.1016/j.bpsgos.2022.05.008 PMC10382704

[22] A. Duffy , J. Horrocks , S. Doucette , C. Keown‐Stoneman , S. McCloskey , and P. Grof , “The Developmental Trajectory of Bipolar Disorder,” British Journal of Psychiatry 204, no. 2 (2014): 122–128. 10.1192/bjp.bp.113.126706 24262817

[23] G. Faedda , R. Baldessarini , C. Marangoni , et al., “An International Society of Bipolar Disorders Task Force Report: Precursors and Prodromes of Bipolar Disorder,” Bipolar Disorders 21, no. 8 (2019): 720–740. 31479581 10.1111/bdi.12831

[24] F. Iorfino , E. Scott , J. Carpenter , et al., “Clinical Stage Transitions in Persons Aged 12 to 25 Years Presenting to Early Intervention Mental Health Services With Anxiety, Mood, and Psychotic Disorders,” JAMA Psychiatry 76, no. 11 (2019): 1167–1175. 31461129 10.1001/jamapsychiatry.2019.2360 PMC6714017

[25] J. Scott , N. Martin , R. Parker , B. Couvy‐Duchesne , S. Medland , and I. Hickie , “Prevalence of Self‐Reported Subthreshold Phenotypes of Major Mental Disorders and Their Association With Functional Impairment, Treatment, and Full‐Threshold Syndromes in a Community‐Residing Cohort of Young Adults,” Early Intervention in Psychiatry 15, no. 2 (2020): 306–313. 32052564 10.1111/eip.12942

[26] E. Brietzke , A. R. Rosa , M. Pedrini , M. N. Noto , F. Kapczinski , and J. Scott , “Challenges and Developments in Research of the Early Stages of Bipolar Disorder,” Brazilian Journal of Psychiatry 38, no. 4 (2016): 329–337. 27533022 10.1590/1516-4446-2016-1975 PMC7111347

[27] G. Salazar de Pablo , A. Cabras , J. Pereira , et al., “Predicting Bipolar Disorder I/II in Individuals at Clinical High‐Risk: Results From a Systematic Review,” Journal of Affective Disorders 15, no. 325 (2023): 778–786. 10.1016/j.jad.2023.01.045 36657494

[28] J. Zhao , Q. Feng , P. Wu , et al., “Learning From Longitudinal Data in Electronic Health Record and Genetic Data to Improve Cardiovascular Event Prediction,” Scientific Reports 9 (2019): 717. 30679510 10.1038/s41598-018-36745-x PMC6345960

[29] D. Chicco and G. Jurman , “The Advantages of the Matthews Correlation Coefficient (MCC) Over F1 Score and Accuracy in Binary Classification Evaluation,” BMC Genomics 21 (2020): 6. 31898477 10.1186/s12864-019-6413-7 PMC6941312

[30] S. D. Nielsen , R. Christensen , T. Madsen , K. Karstoft , L. Clemmensen , and M. Benros , “Prediction Models of Suicide and Non‐Fatal Suicide Attempt After Discharge From a Psychiatric Inpatient Stay: A Machine Learning Approach on Nationwide Danish Registers,” Acta Psychiatrica Scandinavica 148, no. 6 (2023): 525–537. 37961014 10.1111/acps.13629

[31] J. Scott , J. Crouse , N. Ho , et al., “Can Network Analysis of Self‐Reported Psychopathology Shed Light on the Core Phenomenology of Bipolar Disorders in Adolescents and Young Adults?,” Bipolar Disorders 23, no. 6 (2021): 584–594. 33638252 10.1111/bdi.13067 PMC8387492

[32] R. Kessler , J. Abelson , O. Demler , et al., “Clinical Calibration of DSM‐IV Diagnoses in the World Mental Health (WMH) Version of the World Health Organization (WHO) Composite International Diagnostic Interview (WMHCIDI),” International Journal of Methods in Psychiatric Research 13, no. 2 (2004): 122–139. 15297907 10.1002/mpr.169 PMC6878301

[33] B. Milne , A. Caspi , R. Crump , et al., “The Validity of the Family History Screen for Assessing Family History of Mental Disorders,” American Journal of Medical Genetics 150, no. 1 (2009): 41–49. 10.1002/ajmg.b.30764 PMC3750954 18449865

[34] J. Burns , T. Davenport , L. Durkin , G. Luscombe , and I. Hickie , “The Internet as a Setting for Mental Health Service Utilisation by Young People,” Medical Journal of Australia 192 (2010): S22–S26. 20528703 10.5694/j.1326-5377.2010.tb03688.x

[35] B. Mitchell , J. Thorp , Y. Wu , et al., “Polygenic Risk Scores Derived From Varying Definitions of Depression and Risk of Depression,” JAMA Psychiatry 78, no. 10 (2021): 1152–1160. 34379077 10.1001/jamapsychiatry.2021.1988 PMC8358814

[36] T. Wang , X. Zhang , A. Li , et al., “Polygenic Risk for Five Psychiatric Disorders and Cross‐Disorder and Disorder‐Specific Neural Connectivity in Two Independent Populations,” NeuroImage: Clinical 14 (2017): 441–449. 28275544 10.1016/j.nicl.2017.02.011 PMC5328751

[37] J. Witten , D. Hastie , and R. Tibshirani , An Introduction to Statistical Learning (Springer New York, 2013), 49–72.

[38] M. Bracher‐Smith , K. Crawford , and V. Escott‐Price , “Machine Learning for Genetic Prediction of Psychiatric Disorders: A Systematic Review,” Molecular Psychiatry 26, no. 1 (2021): 70–79. 32591634 10.1038/s41380-020-0825-2 PMC7610853

[39] L. Rizkallah , “Enhancing the Performance of Gradient Boosting Trees on Regression Problems,” Journal of Big Data 12 (2025): 35.

[40] K. H. Zou , A. J. O'Malley , and L. Mauri , “Receiver‐Operating Characteristic Analysis for Evaluating Diagnostic Tests and Predictive Models,” Circulation 115 (2007): 654–657. 17283280 10.1161/CIRCULATIONAHA.105.594929

[41] F. S. Nahm , “Receiver Operating Characteristic Curve: Overview and Practical Use for Clinicians,” Korean Journal of Anesthesiology 75, no. 1 (2022): 25–36. 35124947 10.4097/kja.21209 PMC8831439

[42] E. Gentili , G. Franchini , R. Zese , et al., “Machine Learning From Real Data: A Mental Health Registry Case Study,” Computer Methods and Programs in Biomedicine Update 5 (2020): 100132.

[43] J. Scott , G. Murray , C. Henry , et al., “Activation in Bipolar Disorders: A Systematic Review,” JAMA Psychiatry 74, no. 2 (2017): 189–196. 28002572 10.1001/jamapsychiatry.2016.3459

[44] E. Cheniaux , L. Anunciacao , J. Landeira‐Fernandez , and A. Nardi , “Mood or Energy/Activity Symptoms in Bipolar Mania: Which Are the Most Informative?,” Trends in Psychiatry and Psychotherapy 46 (2024): e20220551. 36745539 10.47626/2237-6089-2022-0551 PMC11453170

[45] S. M. Goodday , M. Preisig , M. Gholamrezaee , P. Grof , J. Angst , and A. Duffy , “The Association Between Self‐Reported and Clinically Determined Hypomanic Symptoms and the Onset of Major Mood Disorders,” BJPsych Open 3, no. 2 (2017): 71–77. 28357133 10.1192/bjpo.bp.116.004234 PMC5350540

[46] K. S. O'Connell and B. J. Coombes , “Genetic Contributions to Bipolar Disorder: Status and Future Directions,” Psychological Medicine 51 (2021): 2156–2167. 33879273 10.1017/S0033291721001252 PMC8477227

[47] R. Allesoe , W. Thompson , D. Hougaard , et al., “Deep Learning for Cross‐Diagnostic Prediction of Mental Disorder Diagnosis and Prognosis Using Danish Nationwide Register and Genetic Data,” JAMA Psychiatry 80, no. 2 (2023): 146–155. 36477816 10.1001/jamapsychiatry.2022.4076 PMC9857190

[48] P. Grimes , M. Adams , G. Thng , et al., “Genetic Architectures of Adolescent Depression Trajectories in 2 Longitudinal Population Cohorts,” JAMA Psychiatry 81, no. 8 (2024): 807–816. 38748406 10.1001/jamapsychiatry.2024.0983 PMC11097103

[49] J. Mollon , L. Schultz , E. Knowles , S. Jacquemont , D. Glahn , and L. Almasy , “Low Stability and Specificity of Polygenic Risk Scores for Major Psychiatric Disorders Limit Their Clinical Utility,” Biological Psychiatry 98, no. 6 (2025): 476–484. 40113122 10.1016/j.biopsych.2025.03.006 PMC12353670

[50] Z. Li , D. Li , and X. Chen , “Characterizing the Polygenic Overlaps of Bipolar Disorder Subtypes With Schizophrenia and Major Depressive Disorder,” Journal of Affective Disorders 15, no. 309 (2022): 242–251. 10.1016/j.jad.2022.04.097 35487438

[51] W. Peyrot , G. Panagiotaropoulou , L. Olde Loohuis , et al., “Distinguishing different psychiatric disorders using DDx‐PRS,” medRxiv 7 (2024): 2024.02.02.24302228. 10.1038/s41588-026-02684-x PMC13559851 42625058

[52] E. J. Costello , S. Mustillo , A. Erkanli , G. Keeler , and A. Angold , “Prevalence and Development of Psychiatric Disorders in Childhood and Adolescence,” Archives of General Psychiatry 60, no. 8 (2003): 837–844. 12912767 10.1001/archpsyc.60.8.837

