# Supplementary material for: Can We Improve the Prediction of Early Onset Mania and Hypomania in the Community?
Source: Bipolar Disord. 2026 Jun 25;28(5):e70140. doi: 10.1111/bdi.70140 (PMC13305690; doi:10.1111/bdi.70140)
Supplement: Supplementary file 1 — Appendix 1 STROBE checklist. Appendix 2: Overview of BLTS & further details of assessment tools, analysis and additional references. Appendix 3: Supplementary Tables and Figures. Table S1: Comparison of cases included in the study cohort (n = 1473) with excluded cases (n = 342). Figure S1: Diagrammatic representation of CIDI diagnoses in sample of 1473. Table S2: Sub‐optimal models generated from boosting analyses of risk attributes. [file BDI-28-0-s001.docx]

**Appendices**

**Appendix 1- STROBE checklist**

**Appendix 2- Additional methodological details & Additional references**

**Appendix 3- Supplementary Analyses, Tables and Figures**

**Appendix 1- STROBE checklist**

STROBE Statement—Checklist of items that should be included in reports of ***cohort studies***

|  | Item No | Recommendation | PAGE |
| --- | --- | --- | --- |
| **Title and abstract** | 1 | (*a*) Indicate the study’s design with a commonly used term in the title or the abstract | 1 |
|  |  | (*b*) Provide in the abstract an informative and balanced summary of what was done and what was found | 2 |
| Introduction | | |  |
| Background/rationale | 2 | Explain the scientific background and rationale for the investigation being reported | 3 |
| Objectives | 3 | State specific objectives, including any prespecified hypotheses | 5 |
| Methods | | |  |
| Study design | 4 | Present key elements of study design early in the paper | 4-6 |
| Setting | 5 | Describe the setting, locations, and relevant dates, including periods of recruitment, exposure, follow-up, and data collection | 5-6 |
| Participants | 6 | (*a*) Give the eligibility criteria, and the sources and methods of selection of participants. Describe methods of follow-up | 6-7 |
|  |  | (*b*) For matched studies, give matching criteria and number of exposed and unexposed | NA |
| Variables | 7 | Clearly define all outcomes, exposures, predictors, potential confounders, and effect modifiers. Give diagnostic criteria, if applicable | 5-7 & APPENDIX |
| Data sources/ measurement | 8 | For each variable of interest, give sources of data and details of methods of assessment (measurement). Describe comparability of assessment methods if there is more than one group | 5-7 & APPENDIX |
| Bias | 9 | Describe any efforts to address potential sources of bias | 4-7 |
| Study size | 10 | Explain how the study size was arrived at | 4-7 |
| Quantitative variables | 11 | Explain how quantitative variables were handled in the analyses. If applicable, describe which groupings were chosen and why | 5-7 |
| Statistical methods | 12 | (*a*) Describe all statistical methods, including those used to control for confounding | 8-9 |
|  |  | (*b*) Describe any methods used to examine subgroups and interactions | 8 & APPENDIX |
|  |  | (*c*) Explain how missing data were addressed | 6-8 |
|  |  | (*d*) If applicable, explain how loss to follow-up was addressed | 5-7 |
|  |  | (*e*) Describe any sensitivity analyses | NA |
| Results | | |  |
| Participants | 13 | (a) Report numbers of individuals at each stage of study—eg numbers potentially eligible, examined for eligibility, confirmed eligible, included in the study, completing follow-up, and analysed | 6-8 & APPENDIX |
|  |  | (b) Give reasons for non-participation at each stage | COUVY-DUCHESNE & AUTHORS |
|  |  | (c) Consider use of a flow diagram |  |
| Descriptive data | 14 | (a) Give characteristics of study participants (eg demographic, clinical, social) and information on exposures and potential confounders | 6-10 & APPENDIX |
|  |  | (b) Indicate number of participants with missing data for each variable of interest | COUVY-DUCHESNE & AUTHORS |
|  |  | (c) Summarise follow-up time (eg, average and total amount) | Scott et al 2023 |
| Outcome data | 15 | Report numbers of outcome events or summary measures over time | 8-10 |
| Main results | 16 | (*a*) Give unadjusted estimates and, if applicable, confounder-adjusted estimates and their precision (eg, 95% confidence interval). Make clear which confounders were adjusted for and why they were included | 10-12 |
|  |  | (*b*) Report category boundaries when continuous variables were categorized | 10-12 |
|  |  | (*c*) If relevant, consider translating estimates of relative risk into absolute risk for a meaningful time period | 10-12 |
| Other analyses | 17 | Report other analyses done—eg analyses of subgroups and interactions, and sensitivity analyses | 11-12 & APPENDIX |
| Discussion | | |  |
| Key results | 18 | Summarise key results with reference to study objectives | 10-14 |
| Limitations | 19 | Discuss limitations of the study, taking into account sources of potential bias or imprecision. Discuss both direction and magnitude of any potential bias | 11-15 |
| Interpretation | 20 | Give a cautious overall interpretation of results considering objectives, limitations, multiplicity of analyses, results from similar studies, and other relevant evidence | 11-15 |
| Generalisability | 21 | Discuss the generalisability (external validity) of the study results | 11-15 |
| Other information | | |  |
| Funding | 22 | Give the source of funding and the role of the funders for the present study and, if applicable, for the original study on which the present article is based | 17 & Acknowledgements |

**Appendix 2: Additional methodological details**

*Background Details of Brisbane Longitudinal Twin Study (BLTS)*

Detailed descriptions of the BLTS protocol and procedures (e.g. data encryption and management of anonymized survey information), recruitment strategies, and all the assessments undertaken in different waves are available elsewhere (e.g. Wright & Martin, 2004; Couvy-Duchesne et al, 2018; Mitchell et al, 2019; Scott et al, 2020; Scott et al, 2023). To summarize, this is a community-based cohort study of twins and their non-twin siblings living in the greater Brisbane area who were recruited via media appeals and word of mouth. Participants were included from the age of 12 onwards and recruitment was undertaken continuously between 1992 and 2015, with repeated follow-up waves every three or so years. Ethnically, the cohort reflects the population structure of Queensland at the time of recruitment, with most participants of European ancestry and minorities of predominantly Asian ancestry.

Written informed consent was obtained from potential participants (if aged >=18) or a parent (if aged <18 years). Individuals were excluded if parental report indicated a history of head injuries, neurological or pre-existing psychiatric conditions, substance misuse, and/or taking medications with significant central nervous system effects. An individual twin could be included even if their co-twin was ineligible or declined study participation. Follow-up assessments were coordinated to ensure follow-up waves began with interviews of older cohort members and moved towards younger members. Individuals who missed one follow-up cold be interviewed at the next wave.

As shown in the Figure, more recent follow-ups (waves) have increased the number of ratings of mental health and psychological well-being, etc. The follow-up assessments relevant to this article were undertaken from 2009 onwards (for protocol details see: Couvy-Duchesne et al, 2018; Mitchell et al, 2019). These waves, referred to as 19Up and 25Up, have included mental health assessments such self-report questionnaires about psychological distress and help-seeking and most recently, an assessment of full-threshold mental disorders meeting recognized diagnostic criteria and of family history of mental disorders.

Study Sample

The present study received ethical approval from the Human Research Ethics Committee at the Queensland Institute of Medical Research (QIMR) and is co-ordinated by QIMR Berghofer Institute in Brisbane in conjunction with the Brain and Mind Centre (BMC) at The University of Sydney. De-identified individual data used were extracted from the BLTS dataset according to the following eligibility criteria: the individual had completed an assessment of the self-report rating scales of mental health symptoms (see below) since 2009 and that data regarding CIDI and family history of mental disorders were available from the 19Up or 25Up follow-up respectively. If no CIDI assessment was completed, the individual was ineligible for this study. Also, individuals were excluded from the current project if they had not participated in all the core study assessments and/or the only available symptom self-ratings were recorded after the CIDI assessment (or receipt of a CIDI diagnosis) and/or the timing or sequence of completion of ratings was unclear. Individuals with sporadic missing data (e.g. 1-2 item ratings from the self-report scales or family history assessment) were eligible for inclusion; in these circumstances, missing data were assumed to indicate the absence or negative endorsement of an item (we identified only 13 individuals with missing items scores).


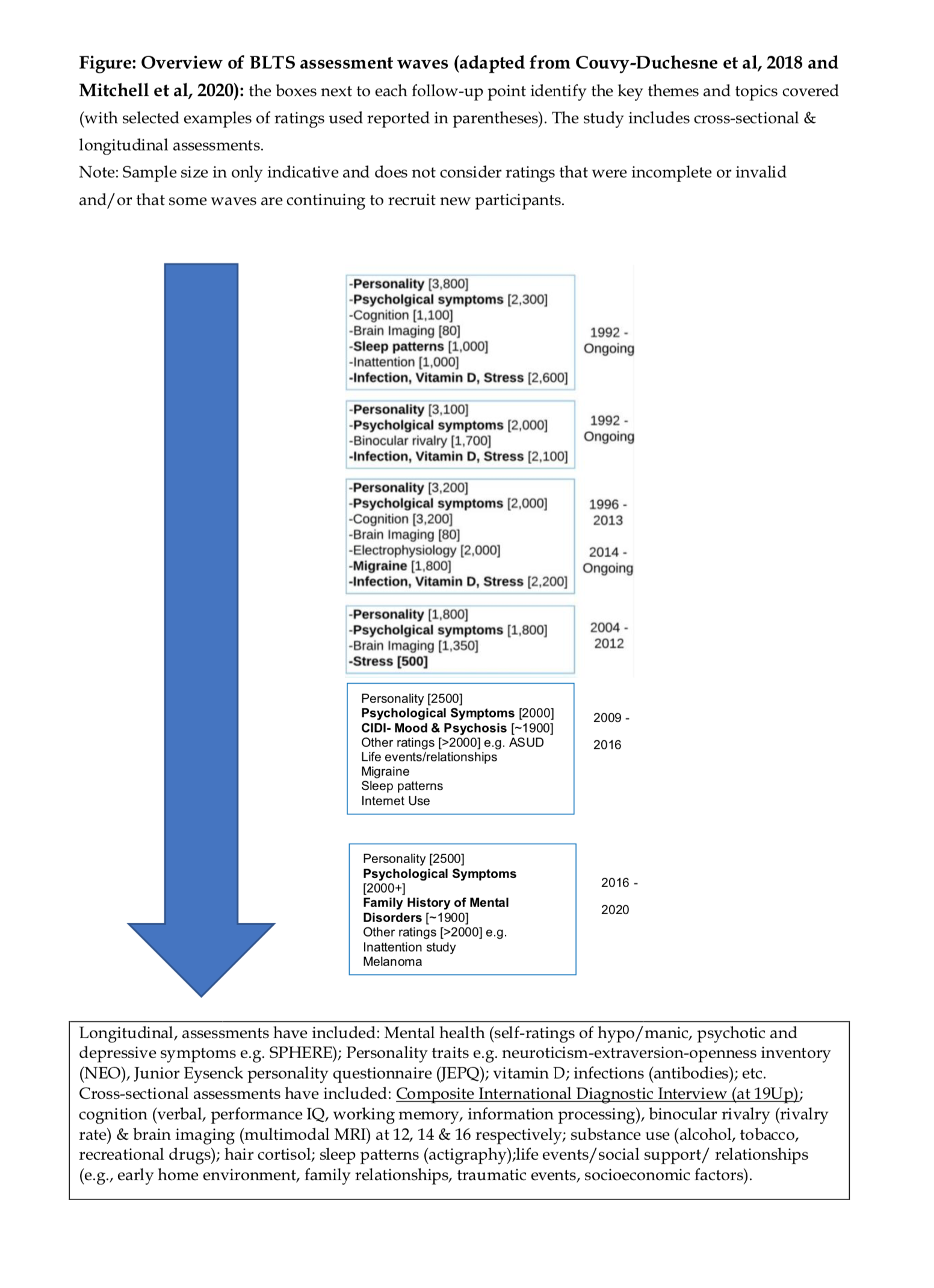


Assessments

1) Demographics: we recorded information regarding age (at completion of CIDI assessment), sex, zygosity, educational status and living situation.

2) Self-Report Ratings of Symptoms and Sub-Threshold Syndromes

The box lists the 23 mood and psychotic symptoms/experiences included in three self-rating scales (Hypomanic-Like Experiences: HMLE; Psychotic Like Experiences: PLE; Depressive-Like Experiences: DLE) [9]. The test-retest reliability of the self-ratings is good (inter-class correlations = 0.8) and the inter-rater reliability (weighted kappas) is about 0.75 for each subthreshold syndrome. These self-report instruments were chosen as they are widely used to evaluate any psychopathology experienced by young people and the ratings can be examined from several perspectives. First, the total number of items endorsed across all three rating scales can be used to estimate overall symptom load (Sx_Load). Second, item endorsements on a particular scale can be used to determine if an individual has experienced a pre-defined subset of symptoms that represent a sub-threshold syndrome (>=6 items for DLE; 5 items for HMLE and >=2 items for PLE).

We extracted data on self-ratings of symptoms and of the associated subthreshold syndromes and noted the age at onset of the latter (or the age of recording of the self-ratings if no subthreshold syndrome was recorded). The assessments include ratings of the following:

1. Hypomanic-Like Experiences (HMLE)
2. Psychotic-Like Experiences (PLE)
3. Depressive-Like Experiences (DLE)

The items rated are shown below:

| **Item Number** | **Items from each scale** |
| --- | --- |
|  | **DLE items** |
| DLE1 | Feel Nervous or Tense |
| DLE2 | Feel Unhappy/Depressed |
| DLE3 | Feel Stressed |
| DLE4 | Feel Overwhelmed |
| DLE5 | Lost Confidence |
| DLE6 | Hopelessness |
| DLE7 | Somatic Pain |
| DLE8 | Hypersomnia |
| DLE9 | Fatigue |
| DLE10 | Impaired Sleep (Quality) |
| DLE11 | Impaired Concentration |
| DLE12 | Anergia |
|  |  |
|  | **HMLE items** |
| HMLE1 | Feel Elated |
| HMLE2 | Increased Self-Esteem/Self-Confidence |
| HMLE3 | Impaired Sleep (Reduced Sleep Need) |
| HMLE4 | Increased Psychomotor Speed (Speech) |
| HMLE5 | Increased Activity (Physical) |
|  |  |
|  | **PLE items** |
| PLE1 | Thoughts Not Your Own |
| PLE2 | Third Party Auditory Hallucinations |
| PLE3 | Heard Voices (when alone) |
| PLE4 | Feel Threatened by Others |
| PLE5 | Paranoia (People are Against You) |
| PLE6 | Thought Withdrawal |

1. Screening for Family History of Mental Disorders

The psychiatric health history of 1^st^ and 2^nd^ degree family members was assessed using an online assessment based on the version of the Family History Screen.

1. Composite International Diagnostic Interview

The CIDI is widely used to determine ‘caseness’ and age at onset for a range of DSM-IV disorders (Kessler et al, 2004). The CIDI was undertaken by interview in the earliest follow-ups of the BLTS, but more recently via an online assessment programme. There are relatively few skip questions, but diagnoses can only be confirmed after further input from researchers who apply a scoring algorithm to the data to allow DSM IV diagnoses to be derived (Couvy-Duchesne et al, 2018). In the current study we focus on mood and psychotic disorders, so we extracted data regarding diagnoses of depressive, manic and hypomanic episodes according to core diagnostic criteria (A and B items of the DSM IV), but without enforcing exclusions (that no longer exist in DSM-5) e.g. bereavement in depression (Scott et al, 2018; ibid, 2020). It should be noted that the CIDI assessment we employed does not evaluate all the psychotic symptoms described in diagnostic criteria for the DSM (e.g. ratings are lacking for negative symptoms). As such, a full threshold psychotic syndrome was defined as present if the individual manifested severe and persistent core positive symptoms (delusions and/or hallucinations).

1. Polygenic Risk Scores-

There are three broad applications of polygenic

analyses in psychiatry: single disorder analyses,

cross disorder analyses and subphenotype analyses

There are three broad applications of polygenic

analyses in psychiatry: single disorder analyses,

cross disorder analyses and subphenotype analyses

Polygenic scores (PRSs) assess the genetic risk of individuals for a disease. All individuals have DNA risk variants for all common diseases, but genetic susceptibility differences between people reflect the cumulative burden of these (Wray et al, 2021). Although PRS are calculated as a weighted count of risk alleles identified in genome-wide association studies (GWAS), estimation methods differ in terms of which DNA variants are included in the score and the weights assigned to them (Lloyd-Jones et al, 2019). Recent research indicates that although the methods that more formally model genetic architecture have similar performance, SBayesR is ranked highly in most comparisons, and has the highest prediction evaluation statistics in most comparisons of psychiatric disorders (Zhou and Zhao, 2021; Zheng et al,2024).

In this study we report PRS for neuroticism (NEU), depression (MDD), bipolar disorder (BD) and schizophrenia (SCZ) that were estimated using the SBayesR approach (with PRS were calculated using summary statistics from recent GWAS or GWAS meta-analyses of psychiatric disorders.)

For PRS estimation, the results of previous large GWAS (discovery sample) were used to calculate an aggregated genetic risk score for each individual in the independent (BLTS) genotyped sample (target sample). SBayesRC is implemented in the publicly available software at <http://cnsgenomics.com/software/gctb> and Illustrations of SBayesR use are provided at <http://cnsgenomics.com/software/gctb/#SummaryBayesianAlphabet>.

Results of the present study were generated from version 2.0 of GCTB. This study also used the following software packages: BayesRv2 at https://github.com/syntheke/bayesR, GCTA (SBLUP, HEreg) (https://cnsgenomics.com/software/gcta/), RSS https://github.com/ stephenslab/rss, LDpred https://github.com/bvilhjal/ldpred, LDSC (version 1.0.0, https:// github.com/bulik/ldsc), PLINK 1.9 http://www.cog-genomics.org/plink/1.9/ and S-PCGC.

Statistical Analyses

In this supplementary material, we add some further details regarding the analyses.

First, we confirm that all analyses were adjusted for age, sex, familiality (including shared environment), zygosity and ancestry. This is especially important given the cohort comprises a large proportion of twins which introduces three sources of liability: additive genetic (A), shared environment (C), and unique environment (E). As such, we addressed these issues through a number of strategies in each analysis. For example, in the preliminary Boosting analysis, we created a single variable of influence that represented all the key elements related to the known liabilities, generating a single predictor variable (i.e., ASAFT) for each individual that represent these different influences/confounders.

Boosting is an ensemble machine learning approach that is useful in imbalanced samples. As shown in the diagram below (adapted from: https:/medium.com/@brijesh_soni/understanding-boosting), Boosting focuses on successively training the basic models in a way that emphasizes misclassified samples from prior iterations. The goal is to prioritize samples that were incorrectly categorized in previous iterations, allowing the model to learn from its mistakes and improve its performance iteratively.

Figure: The Process of Boosting (extracted from: [Understanding Boosting in Machine Learning: A Comprehensive Guide by Brijesh Soni: Medium](https://medium.com/@brijesh_soni/understanding-boosting-in-machine-learning-a-comprehensive-guide-bdeaa1167a6))


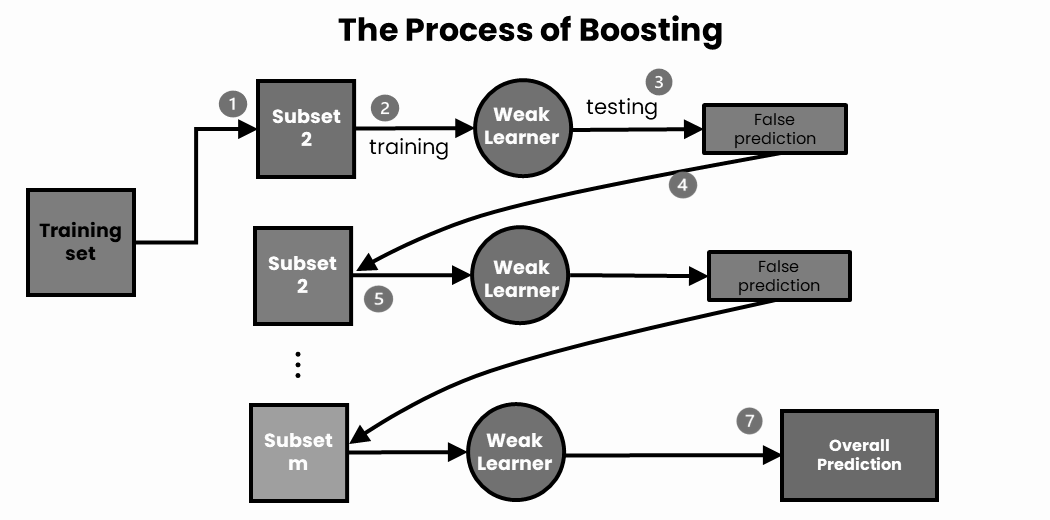


One of the reasons for employing Boosting in this study is because the model combines the predictions from previous iterations, which reduces bias and variance, resulting in more accurate and robust predictions.

As well as allowing estimates of overall accuracy of any classification, the Boosting programme allowed us report the relative influence (or feature importance) and the quality of the binary classification (using the Matthews correlation coefficient: MCC).

Several researchers highlight that feature importance is a useful metric in Boosting analyses as it provides a score that indicates how useful or valuable each attribute was in the construction of the boosted decision trees within the model. The more an attribute is used to make key decisions with decision trees, the higher its relative influence (or feature importance). Feature importance is

calculated for a single decision tree by the amount that each attribute split point improves the performance measure, weighted by the number of observations the node is responsible for. The performance measure may be the purity (Gini index) used to select the split points or another more specific error function. The feature importances are then averaged across all of the decision trees within the model.

The Matthews correlation coefficient (MCC) is calculated using the following formula:

MCC = TP X TN – FP x FN

[(TP=FP)(TP+FN)(TN+FP)(TN+FN)]^1/2^

where:

· TP (true positive) is the number of true positive predictions (i.e., the number of positive cases that were correctly identified as positive).

· TN (true negative) is the number of true negative predictions (i.e., the number of negative cases that were correctly identified as negative).

· FP (false positive) is the number of false positive predictions (i.e., the number of negative cases that were incorrectly identified as positive).

· FN (false negative) is the number of false negative predictions (i.e., the number of positive cases that were incorrectly identified as negative).

As the MCC value is symmetric, the order of the positive and negative classes does not matter.

References

This provides references included in the Appendices and additional references that were excluded from the main text.

Altman EG, Hedeker D, Peterson JL. The Altman Self-Rating Mania Scale. Biol Psychiatry 1997; 42:948–55.

Angst J, Gamma A, Benazzi F, Ajdacic V, Eich D, Rössler W. Toward a re-definition of subthreshold bipolarity: epidemiology and proposed criteria for bipolar-II, minor bipolar disorders and hypomania. J Affect Disord. 2003 Jan;73(1-2):133-46.

Angst J, Cui L, Swendsen J, Rothen S, Cravchik A, Kessler RC, Merikangas KR. Major depressive disorder with subthreshold bipolarity in the National Comorbidity Survey Replication. Am J Psychiatry. 2010 Oct;167(10):1194-201.

Bechdolf A, Ratheesh A, Cotton SM, Nelson B, Chanen AM, Betts J, et al. The predictive validity of bipolar at-risk (prodromal) criteria in help-seeking adolescents and young adults: a prospective study. Bipolar Disord. 2014 Aug;16(5):493-504.

Biggs D, De Ville B, Suen E. (1991) A method of choosing multi-way partitions for classiﬁcation and decision trees. J. Appl. Stat. 18, 49–62.

Carpenter JS, Iorfino F, Cross S, Nichles A, Zmicerevska N, Crouse JJ, Palmer JR, Whitton AE, White D, Naismith SL, Guastella AJ, Hermens DF, Scott J, Scott EM, Hickie IB. Cohort profile: the Brain and Mind Centre *Optymise* cohort: tracking multidimensional outcomes in young people presenting for mental healthcare. BMJ Open. 2020 Mar 29;10(3):e030985.

Clarke TK, Lupton MK, Fernandez-Pujals AM, Starr J, Davies G, Cox S, et al. Common polygenic risk for autism spectrum disorder (ASD) is associated with cognitive ability in the general population. Mol Psychiatry. 2016; Mar; 21(3):419-25.

Coombes B, Markota M, Mann J, Colby C, Stahl E, Talati A, et al. Dissecting clinical heterogeneity of bipolar disorder using multiple polygenic risk scores. Transl Psychiatry. 2020 Sep 18;10(1):314.

Couvy-Duchesne B, O'Callaghan V, Parker R, Mills N, Kirk K, Scott J, et al. Nineteen and Up study (19Up): understanding pathways to mental health disorders in young Australian twins. BMJ Open. 2018; Mar 17; 8(3):e018959.

Depue RA, Krauss S, Spoont MR, Arbisi P. General behavior inventory identification of unipolar and bipolar affective conditions in a nonclinical university population. J Abnorm Psychol. 1989 May;98(2):117-26.

Guiyan N, Zeng J, Revez J, Wang Y, Ge T, Restaudi R, et al (2021). A comprehensive evaluation of polygenic score methods across cohorts in psychiatric disorders. Biol Psych, Nov, 90 (9), 611-620.

Greenland S. Interpretation and choice of effect measures in epidemiologic analyses. Am J Epidemiol. 1987 May;125(5):761-8.

Hickie IB, Davenport TA, Hadzi-Pavlovic D. Development of a simple screening tool for common mental disorders in general practice. Med J Aust 2001;175(Suppl): S10–17.

Iorfino F, Scott EM, Carpenter JS, Cross SP, Hermens DF, Killedar M, et al. Clinical Stage Transitions in Persons Aged 12 to 25 Years Presenting to Early Intervention Mental Health Services with Anxiety, Mood, and Psychotic Disorders. JAMA Psychiatry. 2019 Aug 28. 76(11):1167-75.

Kass G. (1980) An exploratory technique for investigating large quantities of categorical data. Appl. Stat. 29, 119–127.

Kessler RC, Abelson J, Demler O, Escobar JI, Gibbon M, Guyer ME, et al. Clinical calibration of DSM-IV diagnoses in the World Mental Health (WMH) version of the World Health Organization (WHO) Composite International Diagnostic Interview (WMHCIDI) Int J Methods Psychiatr Res. 2004;13(2):122–139.

Lee S, Ripke S, Neale B, Faraone S, Purcell S, Perlis R, et al; Cross-Disorder Group of the Psychiatric Genomics Consortium; International Inflammatory Bowel Disease Genetics Consortium (IIBDGC). (2013) Genetic relationship between five psychiatric disorders estimated from genome-wide SNPs. Nat Genet.;45(9):984-994.

Lejeune A, Le Glaz A, Perron P, Sebti J, Baca-Garcia E, Walter M, et al. Artificial intelligence and suicide prevention: a systematic review. Eur Psychiatry. 2022 Feb 15;65(1):1-22.

Leopold K, Ritter P, Correll C, Marx C, Ozgurdal S, Juckel G, et al. Risk constellations prior to the development of bipolar disorders: rationale of a new risk assessment tool. J Affect Disord. 2012 Feb;136(3):1000-10.

Lewinsohn PM, Shankman SA, Gau JM, Klein DN. The prevalence and co-morbidity of subthreshold psychiatric conditions. Psychol Med. 2004 May;34(4):613-22.

Lloyd-Jones L, Zeng J, Sidorenko J, Yengo L, Moser G, Kemper K, et al. Improved polygenic prediction by Bayesian multiple regression on summary statistics. Nat Commun 10, 5086 (2019).

Milne BJ, Moffitt TE, Crump R, Poulton R, Rutter M, Sears M et al. How should we construct psychiatric family history scores? A comparison of alternative approaches from the Dunedin Family Health History Study. Psychol Med. 2008; 38(12):1793-1802.

Milne B, Caspi A, Crump R, Poulton R, Rutter M, Sears M, Moffitt T. The validity of the family history screen for assessing family history of mental disorders. Am J Med Genet. 2009 Jan 5;150(1):41-9.

Mitchell B, Campos A, Renteria M, Parker R, Sullivan L, McAloney K, et al. Twenty-Five and Up (25Up) Study: A New Wave of the Brisbane Longitudinal Twin Study. Twin Res Hum Genet. 2019 Jun; 22(3):154-163.

Murray GK, Lin T, Austin J, McGrath JJ, Hickie IB, Wray NR. (2021) Could polygenic risk scores be useful in psychiatry? A review. JAMA Psychiatry. Feb 1;78(2):210-219.

Post R, Altshuler L, Kupka R, McElroy S, Frye M, Rowe M, et al. Illnesses in siblings of US patients with bipolar disorder relate to multigenerational family history and patients’ severity of illness. J Affect Disord. 2017 Jan; 207(1):313-319.

Scott J, Graham A, Yung A, Morgan C, Bellivier F, Etain B. A systematic review and meta-analysis of delayed help-seeking, delayed diagnosis and duration of untreated illness in bipolar disorders. Acta Psychiatr Scand. 2022 Nov;146(5):389-405.

Scott J, Martin N, Parker R, Couvy-Duchesne B, Medland S, Hickie I. Prevalence of self-reported subthreshold phenotypes of major mental disorders and their association with functional impairment, treatment and full-threshold syndromes in a community-residing cohort of young adults. Early Interv Psychiatry. 2020. Feb 12. [Epub ahead of print].

Scott J, Davenport TA, Parker R, Hermens DF, Lind PA, Medland SE, Hickie IB. Pathways to depression by age 16 years: Examining trajectories for self-reported psychological and somatic phenotypes across adolescence. J Affect Disord. 2018 Apr 1; 230:1-6.

Scott J, Marwaha S, Ratheesh A, Macmillan I, Yung AR, Morriss R, Hickie IB, Bechdolf A. Bipolar At-Risk Criteria: An Examination of Which Clinical Features Have Optimal Utility for Identifying Youth at Risk of Early Transition from Depression to Bipolar Disorders. Schizophr Bull. 2017 Jul 1;43(4):737-744.

Song J, Pasman J, Johansson V, Kuja-Halkola R, Harder A, Karlsson R, et al. Polygenic risk scores and twin concordance for schizophrenia and bipolar disorder. JAMA Psychiatry. 2024 Dec 1;81(12):1246-1252.

Steadman HJ, Silver E, Monahan J, Appelbaum PS, Robbins PC, Mulvey EP, et al. A classification tree approach to the development of actuarial violence risk assessment tools. Law Hum Behav. 2000; 24(1):83-100.

Tonini E, Crouse J, Shin M, Scott J, Carpenter J, Nichles A, et al. Activation differentiates illness trajectories among youth seeking mental health care. J Affect Disord. 2025 Jun 15; 379:680-689.

Waugh MJ, Meyer TD, Youngstrom EA, Scott J. A review of self-rating instruments to identify young people at risk of bipolar spectrum disorders. J Affect Disord. 2014 May; 160:113-21.

Whitfield JB, Colodro-Conde L, Timmers P, Joshi PK, Montgomery GW, Martin NG. Comparison of Familial, Polygenic and Biochemical Predictors of Mortality. Twin Res Hum Genet.2020; Dec;23(6):307-315.

Winton-Brown TT, Harvey SB, McGuire PK. The diagnostic significance of BLIPS (Brief Limited Intermittent Psychotic Symptoms) in psychosis. Schizophr Res. 2011;131(1-3):256-257.

Wray N, Lee S, Mehta D, Vinkhuyzen A, Dudbridge F, Middeldorp C. Research review: Polygenic methods and their application to psychiatric traits. J Child Psychol Psychiatry. 2014 Oct;55(10):1068-87.

Wray N, Lin T, Austin J, McGrath J, Hickie I, Murray G, Visscher P. From Basic Science to Clinical Application of Polygenic Risk Scores: A Primer. JAMA Psychiatry. 2021 Jan 1;78(1):101-109.

Wright MJ, Martin NG. Brisbane adolescent twin study: Outline of study methods and research projects. Aust J Psychol 2004; 56:65–78.

Yung AR, Nelson B, Baker K, Buckby JA, Baksheev G, Cosgrave EM. Psychotic-like experiences in a community sample of adolescents: implications for the continuum model of psychosis and prediction of schizophrenia. Aust N Z J Psychiatry. 2009;43(2):118-28.

Zhang Z, Zhao Y, Canes A, Steinberg D, Lyashevska O; written on behalf of AME Big-Data Clinical Trial Collaborative Group. Predictive analytics with gradient boosting in clinical medicine. Ann Transl Med. 2019 Apr;7(7):152.

Zhao J, Feng Q, Wu P, et al. Learning from longitudinal data in electronic health record and genetic data to improve cardiovascular event prediction. Sci Rep 2019; 9:717.

Zheng Z, Liu S, Sidorenko J, Wang Y, Lin T, Yengo L, et al. Leveraging functional genomic annotations and genome coverage to improve polygenic prediction of complex traits within and between ancestries. Nat Genet. 2024 May;56(5):767-777.

Zhou G, Zhao H. (2021) A fast and robust Bayesian nonparametric method for prediction of complex traits using summary statistics. PLoS Genet. 17(7): e1009697.

Appendix 3- Supplementary Analyses, Tables and Figures

Page Number

14. Table 1S and Figure 1S

15. Principal Component Analysis of PRS

16. Table 2S

| **Table 1S: Comparison of cases included in the study cohort (n=1473) with excluded cases (n=342)*** | | | |
| --- | --- | --- | --- |
| **Characteristic** | **Current Study Cohort**  **(N = 1473)** | **Excluded from Study**  **Cohort**  **(N=342)** | **X^2^ or t-test**  **signif. (p)** |
| Mean Age in years (with SD) | 26.3 (4.4) | 26.9 (4.7) | 0.08 |
|  | ***Number (%)*** | ***Number (%)*** |  |
| Females | 866 (59%) | 196 (57%) | 0.72 |
| Education: Junior or Senior School only | 267 (18%) | 55 (16%) | 0.1 |
| Full-Time Employment | 869 (59%) | 199 (58%) | 0.39 |
| European Ancestry | 1370 (93%) | 315 (92%) | 0.81 |
| Civil Status: Single | 998 (55%) | 182 (53%) | 0.2 |
| Zygosity: |  |  |  |
| Monozygotic Twins | 427 (29%) | 103 (30%) |  |
| Dizygotic Twins | 530 (36%) | 127 (37%) | 0.42 |
| Non-Twin Siblings | 516 (35%) | 113 (33%) |  |
| *For further details of all the cases (i.e. included and excluded), see Scott et al, PLoS One, 2021,16(6): e0252550.  **Figure 1S: Diagrammatic representation of CIDI diagnoses in sample of 1473** | | | |

**Principal Component Analysis to identify components/dimensions for 4 key PRS being studied**


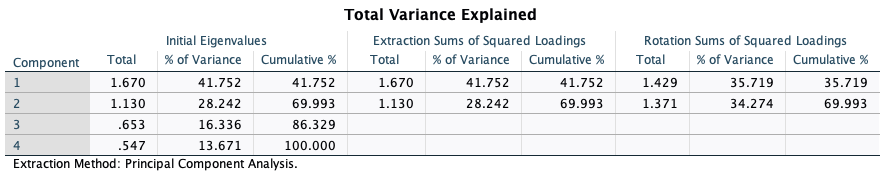


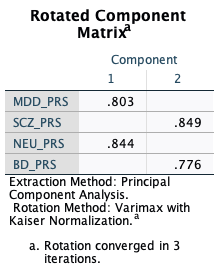


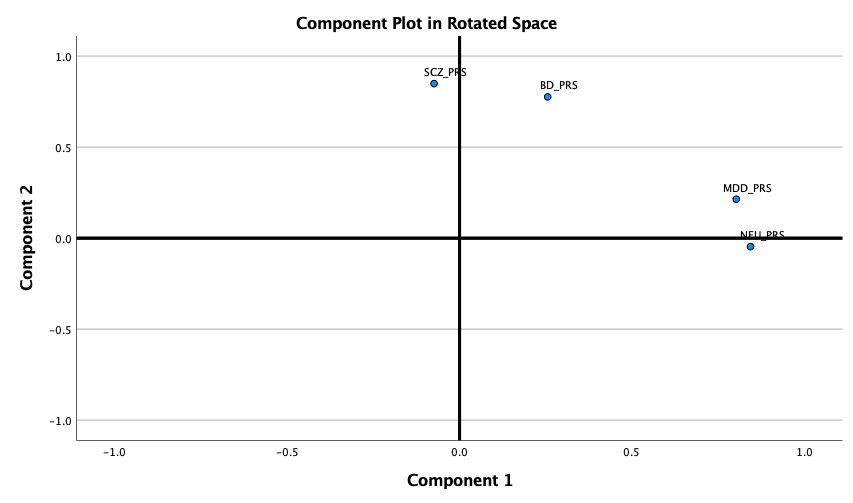


| **Table 2S: Sub-optimal models generated from boosting analyses of risk attributes**  (see main text for details and explanation) | | | | | |
| --- | --- | --- | --- | --- | --- |
|  |  | |  |  |  |
| **MODEL** | **ROC** | |  | **RELATIVE INFLUENCE** |  |
| **Baseline predictor (ASAFT) +** | |  |  | **(feature importance metrics)** |  |
|  |  |  |  |  |  |
| Family History (FH) |  |  |  |  |  |
|  | OVERALL | 0.595 |  | BD | 29.71 |
|  | BD | 0.583 |  | PSY | 13.2 |
|  | No BD | 0.611 |  | MDD | 7.4 |
|  | MCC | .13 |  | MIXED (MOOD & PSY) | 3.25 |
|  |  |  |  |  |  |
|  |  |  |  |  |  |
|  |  |  |  |  |  |
|  |  |  |  |  |  |
| Polygenic Risk Scores (PRS) | |  |  |  |  |
|  | OVERALL | 0.602 |  | BD | 34.56 |
|  | BD | 0.612 |  | SCHIZ | 24.48 |
|  | No BD | 0.594 |  | MDD | 20.53 |
|  | MCC | .19 |  | NEU | 20.48 |
|  |  |  |  |  |  |
|  |  |  |  |  |  |
|  |  |  |  |  |  |
| FH + PRS |  |  |  |  |  |
|  | OVERALL | 0.667 |  | PRS-BD | 29.4 |
|  | BD | 0.676 |  | PRS-MDD | 21.5 |
|  | No BD | 0.658 |  | FH-BD | 19.41 |
|  | MCC | .22 |  | PRS-NEU | 11.32 |
|  |  |  |  | FH-MDD | 6.81 |
|  |  |  |  | PRS-SCHIZ | 5.22 |
|  |  |  |  |  |  |
|  |  |  |  |  |  |
| Symptoms/Syndromes |  |  |  |  |  |
|  | OVERALL | 0.648 |  | DLE | 32.44 |
|  | BD | 0.625 |  | PLE | 24.19 |
|  | No BD | 0.671 |  | HMLE | 15.23 |
|  | MCC | .24 |  |  |  |
|  |  |  |  |  |  |
|  |  |  |  |  |  |
|  |  |  |  |  |  |
|  |  |  |  |  |  |
